# Health-Care Administrator Perspectives on Prevention Guidelines and Healthy Lifestyle Counseling in a Primary Care Setting in New York State

**DOI:** 10.1177/2333392819862122

**Published:** 2019-07-23

**Authors:** Julie Sorensen, Helene Johansson, Lars Jerdén, James Dalton, Henna Sheikh, Paul Jenkins, John May, Lars Weinehall

**Affiliations:** 1 Bassett Healthcare Network Research Institute, Cooperstown, NY, USA; 2 Department of Public Health and Clinical Medicine, Epidemiology and Global Health Unit, Faculty of Medicine, Umeå University, Umeå, Sweden; 3 Center for Clinical Research Dalarna, Falun, Sweden

**Keywords:** primary prevention, health-care guidelines, behavioral counseling, hospital administrators

## Abstract

**Introduction:**

The incidence of chronic disease and treatment costs have been steadily increasing in the United States over the past few decades. Primary prevention and healthy lifestyle counseling have been identified as important strategies for reducing health-care costs and chronic disease prevalence. This article seeks to examine decision-makers’ experiences and self-perceived roles in guideline and lifestyle counseling implementation in a primary care setting in the United States.

**Methods:**

Qualitative interviews were conducted with administrators at a health-care network in Upstate New York and with state-level administrators, such as insurers. Decision-makers were asked to discuss prevention guidelines and healthy lifestyle counseling, as well as how they support implementation of these initiatives. Interviews were analyzed using a thematic analysis framework and relevant sections of text were sorted using a priori codes.

**Results:**

Interviews identified numerous barriers to guideline implementation. These included the complexity and profusion of guidelines, the highly politicized nature of health-care provision, and resistance from providers who sometimes prefer to make decisions autonomously. Barriers to supporting prevention counseling included relatively time-limited patient encounters, the lack of reimbursement mechanisms for counseling, lack of patient resources, and regulatory complexities.

**Conclusions:**

Our research indicates that administrators and administrative structures face barriers to supporting prevention activities such as guideline implementation and healthy lifestyle counseling in primary care settings. They also identified several solutions for addressing existing primary prevention barriers, such as relying on nurses to provide healthy lifestyle support to patients. This article provides an important assessment of institutional readiness to support primary prevention efforts.

## Background

Rates of chronic disease have increased in the United States with an accompanying rise in health-care expenditures over the past few decades. According to the US Centers for Disease Control and Prevention, 50% of US adults suffer from at least one chronic health condition, such as heart disease, cancer, diabetes, or arthritis, while 25% suffer from at least 2 chronic conditions. Nearly half of all deaths in the United States are attributed to heart disease and cancer.^1^

Heart disease, obesity, type 2 diabetes, many cancers, and stroke can often be prevented or ameliorated with exercise, healthy diet, and the elimination of alcohol misuse or tobacco use. Despite the evidence supporting healthy lifestyles, the rates of adult and even childhood obesity continue to climb, with over one-third of US adults reporting a body mass index (BMI) of ≥30 kg/m^2^ and one-fifth of children reporting a BMI ≥95th percentile from 2011 to 2014.^2^ This is at least partially explained by the fact that less than half of US adults engage in the recommended level of daily physical activity.^1^ The prevalence of addictive behaviors also appears daunting, with 20% of adults reporting relatively frequent use of alcohol and tobacco products and 16% reporting binge drinking several times a month.^1^ These modifiable risk factors provide an obvious target for reducing the burden of chronic disease.

Primary care facilities typically serve as the “frontline” in disease prevention. Primary care prevention services, such as counseling to encourage healthy lifestyle changes, have been shown to be effective.^3^ However, the delivery of such services in the United States remains low.^3^ While several barriers have been cited, lack of time has been recognized as a primary obstacle.^4^

Preventive care guidelines, such as those formulated by the US Preventive Services Taskforce (USPSTF),^5^ may facilitate primary care implementation of prevention activities because they provide confirmatory evidence regarding the long-term benefits of primary prevention efforts. Several studies have examined the degree of adherence to USPSTF guideline recommendations in primary care settings, such as prostate cancer screening ^6^ and mammography screening, ^7^ but few have examined adherence to behavioral counseling guidelines and the environmental factors which may affect their implementation (such as administrative support and resource allocation). This is an important question to explore as guidelines are meant to enhance patient care and intensive behavioral counseling for overweight or obese adults and are one of several evidence-based prevention strategies.^5^ Specific USPSTF guidelines that focus on lifestyle and behavioral counseling include alcohol misuse, tobacco smoking cessation, healthy diet, and physical activity.

The authors of this article decided to collaborate on a comparison of prevention guideline implementation and lifestyle counseling in 2 similar regions in Sweden and Upstate New York in order to understand what works and does not work in primary prevention efforts in the primary care setting. These regions include Vasterbotten and Dalarna counties in Sweden and several counties in central New York. These regions are both rural (with some smaller metropolitan areas) and have relatively homogenous populations, and health-care providers in both systems are salaried. The collaboration includes a number of surveys and qualitative assessments with various groups in both health-care systems (ie, patients, physicians, and nurses). Data from these studies have been published elsewhere.^8,9^

Noteworthy findings from this research, however, indicate that physicians in New York more actively promote primary prevention with their patients. More than half of surveyed patients also acknowledged a need to change their physical activity, weight, and eating habits, and 100% indicated a desire to receive support or advice from their primary care provider for these changes.^8^

In addition to physicians and patients, hospital administrators play a key role in primary prevention efforts. Support for this observation is provided by a Spanish study published in 2008.^10^ This study identified several essential components for supporting healthy lifestyle counseling in primary care settings, which included the support of administrators and health-care funders. In particular, recommendations called for “effective administrative support to free practitioners from administrative and bureaucratic tasks” and “agreements between funding bodies and service providers specifically stating health promotion objectives, resources, and indicators for evaluation.” These researchers went on to observe that “current organizational resources in primary health-care centers [are] aimed almost exclusively at caring for disease, [and] make implementation of [healthy lifestyle and prevention] strategies difficult.”^10^

It is necessary to explore how health-care administrators and funding organizations prioritize and support these endeavors in the primary care setting, given the important role they play in the implementation of guidelines and prevention activities. A scan of the public health research literature revealed no published studies that have attempted to explore these questions. This article seeks to fill this gap by examining decision-makers’ experiences and self-perceived roles in guideline and lifestyle counseling implementation in a primary care setting in New York State.

## Method

### Study Setting

Decision-makers were defined as senior administrators in the Bassett Healthcare Network (BHN) system or senior executives in external funding sources, such as insurance companies or Medicaid/Medicare offices. The BHN is an integrated system of hospitals, clinics, long-term care facilities, home care, and medical equipment services that provide a variety of health services to patients residing in an 8-county region of Central New York State. Three of these counties are ranked in the state’s lower half for both health determinants and health outcomes.^11^ The BHN is a not-for-profit organization and its physicians and advanced practice clinicians (APCs) are salaried, with a modest incentive for increased volume. The BHN network was in the process of transitioning to an Accountable Care Organization at the time interviews were conducted, with an anticipated shift in payment structure from a fee-for-service system to one focused on providing high-quality, cost-effective, population-based care. Study recruitment was confined to individuals who met the research team’s definition of “decision-maker,” as described previously. Decision-makers were stratified into 2 groups, which included *organizational* decision-makers, that is, individuals who are responsible for conducting BHN administrative activities, and *environmental* decision-makers, that is, individuals who are responsible for service payment structures, such as insurance companies.

Given the current structure of health-care delivery in the United States, researchers felt that it was important to understand what is happening at both the organizational level and health-care reimbursement level, as both could markedly affect guideline and primary prevention implementation activities. Interviews were structured to elicit information on decision-makers’ efforts and interest in supporting the implementation of prevention guidelines and prevention activities in the patient setting.

### Participant Recruitment

Participants were recruited with the assistance of several physicians connected to the BHN administrative structure and payer networks. Recruiters possessed decades of experience in the BHN and were ideally placed to identify decision-makers who have the ability to develop and facilitate BHN and payer policies. These “key informants” developed a list of organizational and environmental decision-makers and invited these individuals to participate in the study. Invitations were distributed via e-mail and included information about the study, its potential value to the organization, and information regarding what participation would entail. All of the organizational decision-makers who were invited agreed to participate in the study (9 in all). 8 out of 9 organizational decision-makers interviewed serve the BHN as physicians or APCs. Only 2 of the environmental decision-makers responded to our invitation. Both agreed to participate, for a total of 11 interviewees (9 organizational plus 2 environmental decision-makers). One of the environmental decision-makers was also trained as a physician, though he was not currently practicing. Nine of the participants were male, while 2 were female and both environmental decision-makers were male.

### Data Collection

Organizational decision-maker interviews took place in the informant’s office and were conducted by J. Sorensen. For environmental decision-makers, one interview took place in the informant’s office and the other was conducted over the phone. Only the informant and J. Sorensen were present at the time of the interview. Prior to each interview, informants were sent a list of the interview questions and a paper that had been published by the research team, which offered an overview of the study objectives and previous research results. Participants were also told that responses regarding prevention guideline implementation should focus on the USPSTF guidelines specifically. The interview guide was developed by the research team and was constructed according to the research objectives outlined in the original research proposal. In this process, the research team identified a number of key gaps in knowledge relative to decision-maker support for guidelines and primary prevention activities. These gaps in knowledge were then used to formulate a list of questions, which were discussed by the research group and piloted with 3 key informants. These informants, who were not involved in the final interviews, included 2 hospital administrators from the Bassett health-care system and one from the Swedish health-care system. Decision-maker interview questions are provided in Appendix A.

Prior to each interview, informants were given the opportunity to ask questions about the study objectives or to state any concerns regarding interview questions. Interviews were conducted over the course of several months in 2015 by lead author J. Sorensen and only one interview was conducted with each informant. Organizational decision-maker interviews were conducted until new information or insights ceased to emerge (saturation). Unfortunately, this was not possible for environmental decision-makers given the difficulty in accessing this population. All interview audio recordings were transcribed and reviewed by the interviewer for accuracy. Interviews were 45 to 60 minutes in length.

### Data Analysis

The interview transcripts were uploaded into NVivo, a qualitative software program.^12^ Thematic analysis was the guiding analytical framework,^13^ which allowed researchers to summarize the experiences and perceived roles of decision-makers relative to implementing primary prevention guidelines and services in primary care settings. Given this objective, the researchers approached the qualitative inquiry process through the lens of social constructivism, which views reality as both subjective and socially constructed. A priori code categories and subcategories were developed by the research team, using the study objectives for guidance. Developing categories and subcategories in advance allowed the research team to capture transcript sections that were most relevant to the research questions. It also ensured a more efficient coding and sorting process, which involved line-by-line transcript review and coding assignment.

Coding was primarily conducted by the coauthor H. Shiekh of the research team and audited by another team member J. Sorensen. Prior to coding, every manuscript was thoroughly reviewed to provide context and establish familiarity with the data. After coding had been completed for each transcript and reviewed by the auditor, the coder and auditor discussed and further developed category definitions for each of the a priori categories and subcategories. In this way, categories and subcategories were continuously discussed and updated by the coder and auditor based on emerging insights gleaned from transcript reviews. After all transcripts were coded, category summaries were developed for a priori categories; these included information regarding the dimensions and properties of each category. Organizational and environmental decision-maker coding and sorting were conducted separately to permit comparisons between these 2 groups.

### Trustworthiness

Several steps were taken during the process of collecting and analyzing decision-maker interviews to ensure the rigor of the research and the interpretation of the data. To begin, during the coding and categorization process, one author led the coding process while another reviewed these codes against the narrative contained in the transcripts. Differences in opinion were discussed and agreed upon and, if necessary, brought to the larger group of researchers for discussion. Following the development of codes, categories, and category definitions, results were reviewed and discussed by the entire research team who collectively bring over 100 years of experience working within health-care systems (as well, as experience working as administrators). Thus the health-care providers on the research team served to provide “member checks” or “an insider’s perspective” regarding the veracity of the study findings. Lastly, this qualitative study was part of a larger series of studies looking at the implementation of primary prevention and guidelines in primary care settings. These previously conducted surveys and interviews with patients and providers allowed the research team to weigh administrators perspectives against those of patients and health-care providers to identify areas of disagreement, confusion, or discrepancies.

## Results

**Figure 1.**
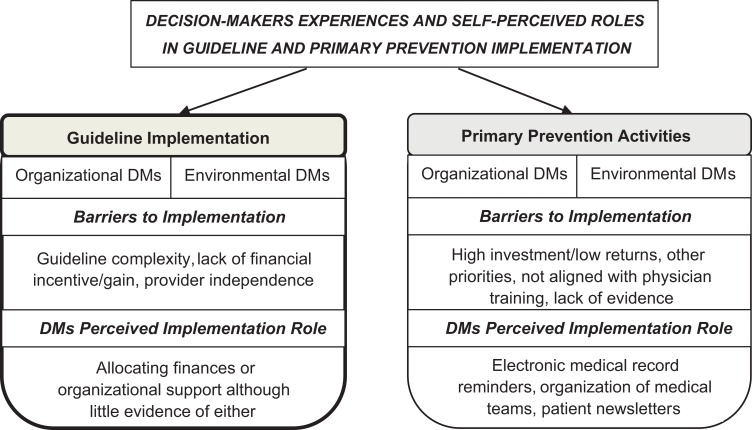
Primary themes identified in decision-maker interviews on guideline and primary prevention implementation.

The aim of this study was to explore how decision-makers prioritize and support guideline implementation and primary prevention activities in the primary care setting. Interestingly, despite the fact that many interview questions were directed at examining decision-makers perceived roles and activities (see “Data Collection” in the Methods section), most of their responses focused on the barriers to supporting these efforts and their lack of a central role in guideline implementation or primary care prevention. As such, summaries of decision-maker responses highlight the primary themes that emerged from the 2 core discussion topics, that is, “healthy lifestyle guideline implementation” and “primary prevention” activities (Figure 1).

### Guideline Implementation

The summary of decision-maker observations relating to this core category feature decision-makers responses to questions about their experiences and self-perceived role in supporting implementation of healthy lifestyle guidelines.

#### Barriers to implementing guidelines

##### Organizational decision-makers

Although every informant was asked to focus his or her response on prevention guidelines relating to physical activity, diet, smoking, and alcohol use, many of the discussions inevitably gravitated to other prevention guideline topics such as cancer screening or maintenance of diabetes. One of the most frequently identified barriers to guideline implementation was the inherent complexity and volume of guidelines. Decision-makers stated that the USPSTF guidelines are just one of many guidelines that providers are asked to follow. At times, informants indicated varying guidelines contradict one another, making it even more difficult to implement them in practice.

At the end of the day, it is very challenging. I don’t want to sound nihilistic about our own responsibility, but I think at some level, just keeping up with the requirements [ie, guidelines], much less setting our own agenda, is challenging.

Decision-makers also observed that many physicians prefer to exercise their own clinical thinking rather than refer to a “cookbook” of clinical directions. From a decision-makers’ perspective, physical activity and diet prevention guidelines can be problematic, as they are not easily reimbursed by payers. These comments imply that responsible investments in primary care prevention could lead to financial losses for health-care facilities.

##### Environmental decision-makers

These decision-makers highlighted the complexity of state and federal rules that reimbursement agencies have to follow as a prominent barrier to prevention guideline implementation. They felt policies and regulations were largely set by various political agendas and special interest groups, impeding the development of a carefully organized and cohesive agenda. They also shared organizational decision-makers’ concerns that complex and seemingly contradictory guidelines can make it difficult for any agency to support guideline implementation. Competition between reimbursement agencies introduces an additional barrier, as supporting prevention guideline implementation could reduce support for other, more lucrative activities.

We are in competition with each other to provide [reimbursement for healthcare], and let us take the exchange. We are all providing the same benefits, so we are competing in quality and price. You…say we are going to cover this preventative benefit at no pocket cost. That increases our cost. It increases the premium cost and, in general, the return on investment for preventive services is not in year one or year two.

#### Decision-maker roles in guideline implementation

##### Organizational decision-makers

Decision-makers indicated that some organizational implementation of guidelines takes place through the electronic medical record (EMR) and the patient-centered medical home model (a team of professionals who work together to provide and coordinate health care across multiple settings). However, they repeatedly pointed out that physicians are in the end responsible for choosing which guidelines to implement and how to do so. There were a few exceptions, such as questioning the patient about smoking, which is considered best practice and highly encouraged by the organization.

Decision-makers did acknowledge that they hold some responsibility for allocating finances or providing organizational-level support for guideline implementation. However, informants offered few examples of specific administrative efforts to implement prevention guidelines. One exception was the mention of initiatives that were also connected to maximizing reimbursement to the institution, such as EMR notices that encourage physicians and patients to follow up on specific prevention recommendations. One example included the organizations efforts to contact patients by phone or e-mail to automatically schedule mammograms and colonoscopies.

Obviously there is a terrific opportunity for social messaging, brochures, materials, reminders, reinforcements, particularly with a fully activated electronic record that I do not think we have begun to optimize.

##### Environmental decision-makers

Environmental decision-makers had very little to say about efforts to implement prevention guidelines. The efforts they did describe related to employees within their own organizations rather than to insured patients. Informants acknowledged that it was possible to provide financial support or other resources. However, few examples were offered in interviews. In general, these informants felt that it was the responsibility of providers to follow recommended practices in treating their patients.

### Prevention Activities

The summary of decision-maker discussions in this core category feature observations about the provision of healthy lifestyle counseling, specifically in relation to diet, physical activity, alcohol use, and smoking cessation in the primary care setting.

#### Barriers to providing healthy lifestyle counseling

##### Organizational decision-makers

Decision-makers felt primary prevention activities were important; however, they also saw many obstacles. Most interviewees felt annual patient checkups offer a good opportunity for physicians to “check-in” with their patients and discuss healthy lifestyle behaviors. However, many lamented that it is difficult for physicians to convince patients to change behaviors. They noted that behavioral change success stories are few and far between. Several stated, “It is an act of faith,” as the success of primary prevention is under the patient’s control, not the provider’s. They also noted that discussions about healthy behavior changes often seem futile given the wider environmental factors that impact patients’ ability to change.

Disease prevention has to do with income inequality, poverty and in a rural area, not having transportation or education. There are so many things that go into health and large chunks of it medical society has nothing to do with.

They also agreed that providing counseling and support for behavior change requires considerable staff time, and when successful, benefits are long term, not immediate. Decision-makers stated that physicians feel conflicted, as there are often more important health issues to address during a patient visit.

Unfortunately, prevention is never really on fire. So sometimes it is difficult for it to get as much traction and attention as other things over all.

Decision-makers also felt that physicians are not the best choice when it comes to promoting healthy lifestyle counseling, as their training focuses on dealing with complex clinical conditions rather than primary prevention. Decision-makers felt prevention counseling was more closely aligned with APC or nurses’ training and experience. Decision-makers also pointed out that keeping people healthy creates a financial dilemma for an institution that makes its revenue from serving sick rather than healthy patients. However, decision-makers also stated this reimbursement system is changing and will likely promote increased dedication to primary disease prevention in its future form. Interviewees did not discuss their perceptions of how family physicians screen for patients in need of primary prevention or how they refer at-risk patients for behavioral counseling.

##### Environmental decision-makers

While environmental decision-makers spoke of using funding to incentivize prevention activities, they identified many barriers to doing so. For example, incentivizing particular patients or employees to pursue healthy lifestyle changes may be perceived as discriminatory. Regulations, such as Health Insurance Portability and Accountability Act and Employee Retirement Income Security Act, impede a payer’s ability to reward patients for health improvements, although specific examples were not provided. The evidence supporting one practice over another is not always clear, which also makes it difficult to decide what to incentivize and what not to incentivize. Finally, they noted that investments in healthy lifestyle changes take a long time to pay off. In general, insurance providers felt they had very little room to maneuver given state mandates.

#### Decision-maker efforts to support primary prevention activities

##### Organizational decision-makers

Decision-makers cited various efforts to support primary prevention activities; however, most of these were physician, APC, and nurse-driven initiatives versus decision-maker-led initiatives. Specific examples of administrative support for prevention activities included a campus-wide ban on smoking and the development of EMR reminders for patient-specific prevention services. They also described sponsoring community activities that promote physical activity or healthy eating and community partnerships to promote the same. However, these appeared to be driven by other individuals or departments in the organization and approved by administrators, versus a central component of an administrator-driven agenda.

Productive strategies that were identified for enhancing prevention efforts were primarily focused on the EMR and the practice of treating patients in medical teams. This strategy allows practitioners with various specialties to do what they do best, while coordinating these activities in ways that best facilitate patient care for at-risk patients. For example, complex clinical activity, such as adjusting multiple medications in a patient with diabetes, heart failure, and chronic kidney disease, can be covered by the physician while nurses, dieticians, or social workers can help patients with questions regarding the day-to-day counseling or application of physician advice. Decision-makers did not discuss the degree to which patients have access to or would use primary prevention programs, such as specialized counseling, patient education, and health promotion programs.

##### Environmental decision-makers

Environmental decision-makers likewise identified few existing efforts to support patients in healthy lifestyle improvements. Efforts that were mentioned included the distribution of patient newsletters providing information on healthy lifestyle practices. Some mailings appear to be tailored to the patient, such as diabetics or asthma patients, covering topics that are specific to controlling these medical conditions. New mandates regarding the health of women and children have led to the reduction in copays for some preventive services. Decision-makers also stated that their employees have free access to fitness centers and receive a health score, so improvements in scores can be rewarded. One decision-maker felt there was considerable evidence that smoking cessation is extremely beneficial and that financial incentives relating to this lifestyle change can lead to positive results.

## Discussion

Implementation of new practices or clinical practice guidelines in health care is often described as complex and challenging. This is the case even when there is an important need for change. A multilevel approach for changing clinical practice has been emphasized in the literature,^14,15^ which describes different frameworks or determinants that influence both the implementation process and outcomes.^15-18^ These frameworks share similarities that include 4 determinant domains, which are commonly represented. These include (1) characteristics of the implementation object, (2) characteristics of the adopters (ie, health-care practitioners), (3) system/organizational context (inner and outer), and (4) implementation strategies.^19,20^

### Main Findings

One of the primary findings in our discussions with decision-makers, both organizational and environmental, is that, while they believe primary prevention is a worthy endeavor, they experience significant barriers to supporting primary prevention in the primary care setting, as reflected^21^ in the domain “system/organizational context.” These barriers include a lack of support and resources at the organizational level, deriving from a lack of resources coming from state and federal health-care decision-makers, as well as the pressures of state and federal regulations and marketplace competition at the environmental level. This finding is in line with other studies indicating that the sociopolitical context and the wider macro system environment can exert strong influence on implementation outcomes and an organizational capacity to change.^22,23^

In general, organizational decision-makers tended to revert to their roles as clinicians in discussing guidelines and prevention activities. They discussed their frustrations with efforts to encourage primary prevention as physicians, noting, for example, that they have little time with their patients and little training in primary prevention counseling. These perceived barriers are commonly reported in the literature.^21,24-30^

Increasing provider access to continuing education on primary prevention has been a noted gap and potential priority for improving population health in other studies.^16^ Discussions of healthy living also appeared to be incongruous with the realities of their patient’s lives. Doubts about the effectiveness of lifestyle interventions have been identified elsewhere as a common obstacle for the uptake in health-care practices.^21,28,29^ Additionally, Geense et al ^29^ have identified 6 different types of general practitioner based on their attitudes to discussing lifestyle changes with patients. These types span from being an ignorer to a nurturer. Whereas the ignorer believes it is the task of the government to promote a healthy lifestyle, the nurturer perceives the role of the general practitioner as similar to a teacher, meaning physicians should focus efforts on educating their patients.

Conversations regarding prevention activities and guidelines in our study often gravitated toward discussions of procedures that are reimbursable or standard treatment for chronic diseases, such as checking hemoglobin A_1__C_ levels in diabetics. These activities are likely facilitated by decision-makers, through resources such as EMR prompts and predetermined standards for treatment, because reimbursement is linked to hitting A_1__C_ targets. However, as indicated by decision-makers, doctors have a great deal of autonomy in how they treat their patients and many primary prevention procedures are not reimbursed. This may provide some explanation for the paucity of examples highlighting administrative support for primary prevention activities and guideline implementation.

In addition, providers and decision-makers are often faced with multiple sets of guidelines regarding health maintenance and prevention. These are all to some extent “evidence based”; however, despite the evidence, not all guidelines come with the same set of recommendations. In the United States, while the USPSTF is widely respected as an evidence-based and minimally biased organization, there is no one recommended system of guidelines. This may lead to confusion and ambiguity on the part of providers and decision-makers.

The character of the guideline (ie, the implementation focus) has also been identified in the literature as a crucial factor for successful implementation. If the intervention is perceived as clear by stakeholders and target users, is easily trialed, and does not require specific resources, it has a greater chance of being implemented. Additional important factors include the guideline source, the evidence strength and quality, the relative advantage of the new practice (better and worth the cost), and the medical field associated with the guideline.^18,31,32^ For example, an implementation study conducted in primary care by Kardakis et al showed limited physician uptake and usage of clinical practical guidelines on lifestyle interventions, as compared to nurses.^30^ This study’s finding indicates that physicians see less value in guidelines on lifestyle interventions; however, the study recognized other possible factors that impact uptake, such as “guideline fatigue.”^19^

Facilitation has been described as the enabling of the mobilization of knowledge into practice. It relies on a designated facilitator, who can encourage others to reflect on their current practices in order to identify gaps in performance, introduce change, and decisively impact service provision outcomes.^19,33^ Discussions with both organizational and environmental decision-makers in our study appear to indicate that they feel more like followers than leaders or facilitators in the current health-care system, at least in the area of allocation of resources for preventive services. Decision-makers consistently referenced predetermined reimbursement schemes and mandates about what care to provide. Both groups felt there was relatively little room to innovate or create substantive changes in health-care delivery, despite growing evidence that primary prevention is a sound investment.^17^ However, many were also optimistic at the time of their interviews that the Affordable Care Act (ACA) would shift the focus of health care away from reactive disease management toward more proactive health promotion. In general, decision-makers appeared to adopt a “sit and wait” approach to primary prevention rather than a proactive “let us fix it now” response. However, it is important to note that these interviews were conducted in 2015, prior to the proposed 2017 elimination of the ACA and subsequent challenges.

Implementation of guidelines and lifestyle interventions in clinical practices brings unique challenges that are distinctively different from those found in more traditional fields of medicine.^22^ The most significant difference is that primary prevention and health promotion is not an urgent priority.^22,27^ As indicated by both organizational and environmental decision-makers, “traditional” medical care is most frequently prioritized and changing this could be challenging, especially given the long time span between intervention and improved health outcomes. Prolonged investments in long-term outcomes can be troublesome for organizations and policymakers who are often pressured to demonstrate results in relatively short time frames.^22^

In addition to these observations, this summary of decision-maker comments offers helpful suggestions for supporting prevention activities in primary care. Most importantly, decision-makers appear convinced that healthy lifestyle counseling is most closely aligned with APCs, nurses, dieticians, or physiotherapists expertise and most affordably offered by these members of the clinical staff, a theme that has been identified in similar studies.^18^ However, to provide this type of support, facilities would in return require reimbursement and financial support for these investments, a solution which has been previously noted in the primary prevention literature.^16^ Reimbursement strategies will also need to shift from a focus on procedures and disease management toward health promotion, which has been suggested in other peer-reviewed publications, as well.^16^ Finally, environmental decision-makers will need to see evidence that these long-term investments are better than short-term, quick fixes.

### Limitations

The purpose of this study was to use a qualitative framework to explore decision-makers self-perceived roles and experiences with guideline implementation and primary prevention activities in the primary care setting. Thus, what has been gained in the provision of details and nuanced informant perspectives is lost in the ability to gather responses from many participants, thus impacting the generalizability of results. Notably, informant responses may not represent the views or experiences of all administrators or payers who practice in considerably different health-care settings. However, this limitation can be addressed through further qualitative studies that explore decision-makers’ prevention and guideline implementation experiences in other settings. Additionally, given the politicized nature of insurance and Medicaid and the extensive responsibilities given to environmental decision-makers working in these systems, it was very difficult to reach environmental decision-makers to assess their interest in being interviewed. This could also potentially impact the generalizability of comments made by these individuals in our study. However, given the data presented here, future quantitative studies based on these observations may provide an opportunity to test our results. Despite these limitations, there are also several noteworthy strengths. These include the regional and disciplinary diversity of the research team, the relatively widespread representation of decision-makers from the participating health-care network, the high representation from key organizational staff for the health-care facility under study, and the relative lack of data on administrators’ perceived roles in primary prevention activities in the published literature.

## Conclusions

The findings presented in this study contribute to an understanding of decision-makers’ barriers and observations regarding implementing prevention guidelines and healthy lifestyle counseling in the primary care setting. Overall, BHN administrators viewed primary prevention as a valuable goal. However, they cited numerous barriers to pursuing this goal, most prominently a misalignment of financial incentives, limited time, and more urgent health priorities. Our research supports a growing body of evidence that demonstrates the current approach to health care emphasizes reacting to disease rather than disease prevention. Notably, in thinking about prevention, physician decision-makers tended to reflect on their experiences as clinicians versus their role as administrators. This may reflect an environment in which physicians function relatively autonomously in regard to prevention, even within an integrated health-care system with a salary model. Furthermore, decision-makers’ failure to speak from their administrative experience may indicate a perceived lack of agency when it comes to making administrative decisions that can support the prevention agenda. In conclusion, this study illustrates important points about prevention in the primary care setting and merits further qualitative and quantitative investigation in a variety of settings to validate its findings.

## Appendix A

### Moderator’s Guide Questions

#### Guidelines

These questions will relate to the following healthy lifestyle activities: physical activity, healthy diet, and reductions or elimination of tobacco and alcohol use

- Are guidelines used to improve patient care and patient outcomes? If so, how?
- Whose responsibility is it to implement guidelines in patient care?
- What are your experiences working with guidelines? Are they effective/not effective? In what ways?
- Could you discuss any formalized structure for implementing guidelines at BHN?
- How are resources allocated for implementing guidelines?
- Does your organization experience barriers to guideline implementation? If so, what are these?
- Is financial compensation linked to guideline implementation? If so how? If not, should it be and why?

#### Primary prevention activities

These questions will relate to the following healthy lifestyle activities: physical activity, healthy diet, and reductions or elimination of tobacco and alcohol use

- How does your organization facilitate disease prevention activities?
- What role does your organization feel it plays in disease prevention?
- Who in your organization decides whether prevention activities are supported or adopted?
- How are these decisions implemented within your organization?
- What activities does your organization currently engage in to support disease prevention?
- Are disease prevention activities effective (why/or why not)?
- In the delivery of health care, which groups play the most vital role in disease prevention?
- Is financial compensation linked to healthy lifestyle counseling? If so how? If not, should it be and why?
